# Faecal microbiota transplantation in Crohn’s disease: an Australian randomised placebo-controlled trial protocol

**DOI:** 10.1136/bmjopen-2024-094714

**Published:** 2025-04-19

**Authors:** Sasha R Fehily, Emily K Wright, Chamara Basnayake, Amy L Wilson-O’Brien, Annalise Stanley, Elise P Marks, Erin E Russell, Amy L Hamilton, Robert V Bryant, Sam P Costello, Michael A Kamm

**Affiliations:** 1 Department of Gastroenterology, St Vincent’s Hospital Melbourne, Fitzroy, Victoria, Australia; 2 Department of Medicine, The University of Melbourne, Melbourne, Victoria, Australia; 3 Department of Gastroenterology, The Queen Elizabeth Hospital, Woodville South, South Australia, Australia

**Keywords:** Inflammatory bowel disease, Gastroenterology, Microbiota, Clinical trials

## Abstract

**Introduction:**

The enteric microbiota drives inflammation in Crohn’s disease. Yet, there are no placebo controlled trials evaluating the efficacy and safety of faecal microbiota transplantation (FMT) in inducing and maintaining remission in patients with active Crohn’s disease. The Microbial Restoration (MIRO) study aims to establish this evidence.

**Methods and analysis:**

At two specialist inflammatory bowel disease centres, 120 enrolled patients will have a 3-week period of diet optimisation (removal of ultra-processed foods) together with a 7-day course of antibiotics (to facilitate subsequent FMT engraftment). Patients will then be stratified to upper gut (for disease proximal to the splenic flexure) or lower gut (distal to the splenic flexure) disease. Patients will then be randomised in a 2:1 ratio to receive anaerobically prepared stool or placebo for 8 weeks either by gastroscopy, or colonoscopy and enemas. Clinical response at 8 weeks (Crohn’s Disease Activity Index (CDAI) reduction ≥100 points or to <150 points) is the primary outcome measure. Non-responders to placebo and partial responders to FMT (CDAI decrease <100 but >70) receive FMT for weeks 8–16.

Patients achieving clinical response from FMT after 8 or 16 weeks will be randomised in a 1:1 ratio to either a 44-week maintenance phase of FMT or placebo. Patients will receive FMT from one donor throughout the study.

The MIRO study will establish whether FMT is an effective and safe therapy to induce and maintain remission in patients with active Crohn’s disease.

**Ethics and dissemination:**

Ethical approval has been received by the St Vincent’s Hospital Melbourne Human Research Ethics Committee (HREC-A 084/21). The results will be disseminated in peer-reviewed journals and presented at international conferences.

**Trial registration number:**

ClinicalTrials.gov: NCT04970446; Registered on 20 July 2021.

**STRENGTHS AND LIMITATIONS OF THIS STUDY**

- This is a randomised placebo-controlled study, providing the highest level of evidence about both efficacy and safety of faecal microbiota transplantation (FMT) in Crohn’s disease for the induction and maintenance of remission.
- The optimisation phase of diet and antibiotics is postulated to enhance FMT engraftment.
- The scientific analyses will determine organisms, and metabolic pathways, associated with response or lack of response to FMT.
- Confining the study to two tertiary centres will ensure robust compliance with the study protocol, while the diverse patient population ensures generalisability.
- The use of the Crohn’s Disease Activity Index as the primary endpoint, while providing a standard means for establishing comparability with other studies, is inherently largely subjective and therefore potentially unreliable.

## Introduction

### Background and rationale

 Crohn’s disease inflammation is believed to be driven by a perturbed microbiota, influenced by genetic and environmental risk factors.^1^,^3^ Luminal and mucosa-associated Crohn’s disease microbiota profiles are distinctly different from healthy controls and patients with ulcerative colitis (UC).^1^,^8^

Considering the central role of a disturbed microbiota in inflammatory bowel diseases (IBD), faecal microbiota transplantation (FMT) has been postulated to shift the microbiota into a healthy, well-balanced community of organisms, thereby correcting the proinflammatory milieu. Consequently, randomised controlled trials have established the efficacy of FMT in achieving and maintaining remission in active UC.^9^,^18^

A strong motivation for exploring the therapeutic value and safety of FMT in Crohn’s disease relates to the benefits and risks of traditional immunosuppressive therapies, which are associated with clinical response rates of less than 50% by week 12, diminishing response over time,^19 20^ and significant risks.^21^

FMT has shown promising efficacy in Crohn’s disease cohort studies. However, only two randomised controlled trials have been published, with only one including a placebo control group.^22^,^24^ Sokol *et al* performed a small multicentre randomised, single-blind placebo-controlled trial in France (n=17), evaluating the efficacy of a single FMT following clinical remission induced by corticosteroids. Of 17 patients with mainly ileocolonic disease, 8 patients received single donor, fresh stool via colonoscopy and 9 patients received Sham FMT. Corticosteroid-free remission was seen in 88% of the FMT arm versus 44% sham FMT at week 10 (not statistically significant). Endoscopic disease activity (Crohn's Disease Endoscopic Index of Severity) significantly declined by week 6 following FMT (8.5 [4.6; 13.0] vs 3.5 [1.0; 8.9], p=0.03) but not sham treatment (2.4 [0.0; 8.3] vs 2.7 [0.7; 10.0]; p=0.8).^23^

The nature of faecal transplantation used may have bearing on its effectiveness. Whole stool that is anaerobically processed preserves bacterial populations compared with preparation in ambient air.^25^,^27^ The latter is not necessary to achieve cure in *Clostridioides difficile* infection, but may be critical in Crohn’s disease, in which there is a reduced abundance of butyrate-producing oxygen-sensitive organisms.^28^ This Microbial Restoration (MIRO) study will use anaerobically prepared stool.

In the MIRO study, an ‘optimisation’ phase is designed to improve microbial engraftment. In this randomised, double-blind, placebo-controlled trial, the cohort will be stratified into two groups according to disease phenotype to determine route, dose and frequency of FMT administration. Randomisation will be 2:1 for the active intervention group versus control in the induction phase, followed by re-randomisation at 1:1 for the maintenance phase. The study protocol adheres to the Standard Protocol Items: Recommendations for Interventional Trials statement (2013) (online supplemental file 1).

### Objective

This study aims to investigate the efficacy and safety of FMT in inducing and maintaining remission in Crohn’s disease.

## Methods and analysis

### Study setting

Patient diversity will be ensured by the recruitment process. The study will be conducted at two Australian secondary and tertiary specialist IBD centres that serve large and diverse catchment areas. Patients will be referred by gastroenterologists from within, and outside of, these two specialist centres, including from both private practices and public clinics across two Australian states. Study was started in May 2022 with intended conclusion in April 2026.

### Recipient eligibility: induction phase

#### Inclusion criteria

- Participants aged 18–70 years with Crohn’s disease proven by endoscopic, histological and/or radiological assessment.
- Confirmed endoscopic active inflammation (unless isolated small bowel disease that is inaccessible by endoscopy, in which case sonographic inflammation is sufficient) within 6 months of study entry.
- Crohn’s Disease Activity Index (CDAI) Score of 220–450.
- At least one of the following:
  - C-reactive protein (CRP)≥5 mg/L.
  - Faecal calprotectin≥100 µg/g.
  - Inflammation on imaging (either intestinal ultrasound (IUS) or Magnetic Resonance Imaging (MRI).
- Willing and able to attend the study sites for regular endoscopic procedures.

#### Exclusion criteria

Exclusion criteria include active perianal or fistulising disease; pregnant or intending to become pregnant within 12 months; enteropathy or colitis other than Crohn’s disease; symptomatic intestinal stricture likely to require surgical or endoscopic treatment; presence of a stoma; presence of an ileoanal pouch; total white cell count less than 3.0×10^9^/L; albumin less than 20 g/L; immunodeficiency (beyond that caused by immunosuppressants used for the treatment of IBD) for example, HIV or common variable immune deficiency; anaphylaxis/severe allergy to food; thiopurine, methotrexate or biologic agent whose dose has been modified within the past 2 months; small molecule inhibitors whose dose has been modified within 1 month; aminosalicylates use within 2 weeks of study entry; prebiotic, probiotic or antibiotic therapy, or over-the-counter supplement therapy in the 2 weeks prior to study entry; rectal topical Crohn’s disease therapy in the 2 weeks prior to study entry; prednisolone dose >20 mg or budesonide dose >6 mg; unwilling or unable to taper corticosteroids to zero within 8 weeks of initial FMT; active gastrointestinal infection; alcohol consumption of a dependent nature; primary sclerosing cholangitis; any condition that the treating gastroenterologist deems to pose a theoretical risk to the patient undertaking FMT; any patient that the treating clinicians feel is incapable of participating in the safe use of FMT.

### Recipient eligibility: maintenance phase

#### Inclusion criteria

Patients who have had a clinical response (CDAI decrease of ≥100 points or CDAI <150 points) to blinded FMT therapy at week 8, or to open-label FMT at week 16, can enter the maintenance study.

### Donor eligibility

#### Donor inclusion and exclusion criteria

Healthy people aged 16–60 years old who reside within the metropolitan area of the collection centre with a body mass index between 18.5 and 25. Extensive medical history and investigations must exclude the presence of any active gastrointestinal symptoms, medical or psychiatric comorbidities, infectious diseases or antibiotic-resistant bacterial colonisation. Donors must be non-smokers and not use medications, alcohol or recreational drugs.

The donor inclusion and exclusion criteria are defined by the stool bank, BiomeBank, under the jurisdiction of the Australian Therapeutic Goods Administration (TGA). The inclusion and exclusion criteria are regularly examined and amended by a team of gastroenterologists and infectious diseases physicians to reflect the current relevant risks to the population.

### Screening processes

#### Participant screening

Referred patients will be assessed by the trial team for eligibility by an initial phone screen evaluating recent endoscopy results, faecal (calprotectin) and serum (CRP) disease activity biomarkers and relevant imaging. The CDAI Score will be calculated, and medical history taken to identify exclusion criteria. Patients who satisfy eligibility criteria will then be invited to the trial hospital sites for a screening visit.

At the study centre screening visit, an extensive medical history will be taken by a physician and clinical nurse. Venepuncture for routine pathology and infections, and a stool assessment for infectious pathogens, antibiotic-resistant bacteria and faecal calprotectin, is undertaken. An IUS is performed to evaluate the presence of sonographic inflammation (defined by a combination of bowel wall thickness ≥3 mm and hyperaemia (Limberg score ≥2). If the location of disease is inaccessible on IUS, or the views are suboptimal, an MRI scan of the small and large bowel is performed. The same modality of imaging will be conducted for the duration of the trial.

#### Donor screening

The screening assessment involves specialised history taking to identify comorbidities and a family history of medical conditions. Respiratory, blood and stool specimens are obtained to identify medical conditions, chronic infections and colonisation with antibiotic-resistant bacteria.

If the donor meets eligibility, the stool is quarantined for 3 months. The donor is reassessed at 3 months; if re-evaluation is negative, then stool can then be used and the donor qualifies for ongoing donation.

### Randomisation and stratification

Eligible patients will be stratified according to their historical maximal extent of intestinal involvement. Patients with intestinal disease proximal to the splenic flexure will be assigned upper gastrointestinal delivery of therapy. Patients with disease distal to the splenic flexure, or an ileorectal or ileosigmoid anastomosis, will receive lower gastrointestinal delivery.

There will be a total of four randomisation tables. Each table will represent stratification according to the assignment to upper or lower gastrointestinal delivery, and the phases of the study (induction or maintenance). Randomisation within these tables is computer generated by a statistician and then uploaded to a web-based platform.

For the induction phase, patients are randomised to FMT therapy or placebo in fixed block sizes of three patients in a ratio of 2:1, respectively. Re-randomisation occurs for participants entering the maintenance phase, from either the induction or open-label extension phases, in blocks of two in a ratio of 1:1.

### Sample size

The study aims to enrol 120 patients with a goal of 96 patients completing the induction phase. This is based on registration studies evaluating induction of remission with anti-tumour necrosis factor therapy in Crohn’s disease (chimeric monoclonal antibody cA2 study^29^ and CLASSIC I study^30^). Differences ranging from 24% to 29% were achieved (remission rates in intervention group of 33%–36%; remission rates in placebo group of 4%–12%). In addition, two small controlled trials evaluating FMT in Crohn’s disease reported remission rates in the intervention group of 36% and 44% at weeks 8 and 10, respectively.^23 24^

The predicted proportion of patients achieving a response is 60% in the FMT group and 30% in the control group (estimated delta of 30%), based on a double-sided alpha value of 0.05. This also takes into account 20% attrition in each group, to achieve a non-dropout number of 96 patients completing the induction phase of the study.

### Consent

Written and verbal information on the FMT process and informed consent forms will be provided to each patient by the site investigator prior to the screening visit (online supplemental file 2). Written consent for participation in the study will be obtained at the screening visit prior to the collection of medical information and biospecimens. Clinical data will be collected out to 5 years.

Further written consent will be documented at the time of each procedure consenting to the risks associated with the procedure itself, the administration of FMT or placebo, study medications and anaesthesia.

### Primary and secondary endpoints

The primary and secondary endpoints are detailed in table 1.

**Table 1:** Primary and secondary endpoints

Outcome	Definition	Timepoint
Primary		
Clinical response	CDAI decrease of ≥100 or CDAI<150	Week 8
Secondary		
Clinical remission	CDAI<150	Week 8 and week 52Week 16 and week 60 (for open FMT group)
Endoscopic response	SES-CD reduction by 25% and 50%	Week 8 and 52Week 16 and 60
Endoscopic remission	SES-CD ≤2 or absence of ulcers	Week 8 and week 52Week 16 and 60
Histological remission	The absence of ulcers; the absence of acute inflammation histologically; one of either Geboes Score or Robarts Histology Index	Week 8 and 52Week 16 and 60
Radiological remission	IUS (BWT<3 mm and/or Limberg 0 or 1) or MRI (wall thickness<4 mm and no or minimal wall enhancement)	Week 8 and 52Week 16 and 60
Biochemical response	Normalisation of CRP and faecal calprotectin (<50 µg/g, <100 µg/g, <150 µg/g, <200 µg/g, <250 µg/g	Week 8 and 52Week 16 and 60
Time to outcomes	Time taken to achieve clinical response or remission during induction and maintenance phases	Duration of trial
Maintenance of clinical remission	CDAI <150	Weeks 52 or 60
Sustained clinical remission	CDAI <150	Weeks 52 or 60
Steroid-free clinical remission		Weeks 52 or 60
Safety outcomes	Adverse events	Duration of trial
Scientific outcomes	Comparison of genetic, microbiological, metabolic and immunological factors in the responders with the non-responders	Week 8 and 52Week 16 and 60

BWT, bowel wall thickness; CDAI, Crohn’s Disease Activity Index; CRP, C reactive protein; FMT, faecal microbiota transplantation; IUS, intestinal ultrasound; SES-CD, simplified endoscopic activity score for Crohn's disease.

### Study design

**Figure 1:**
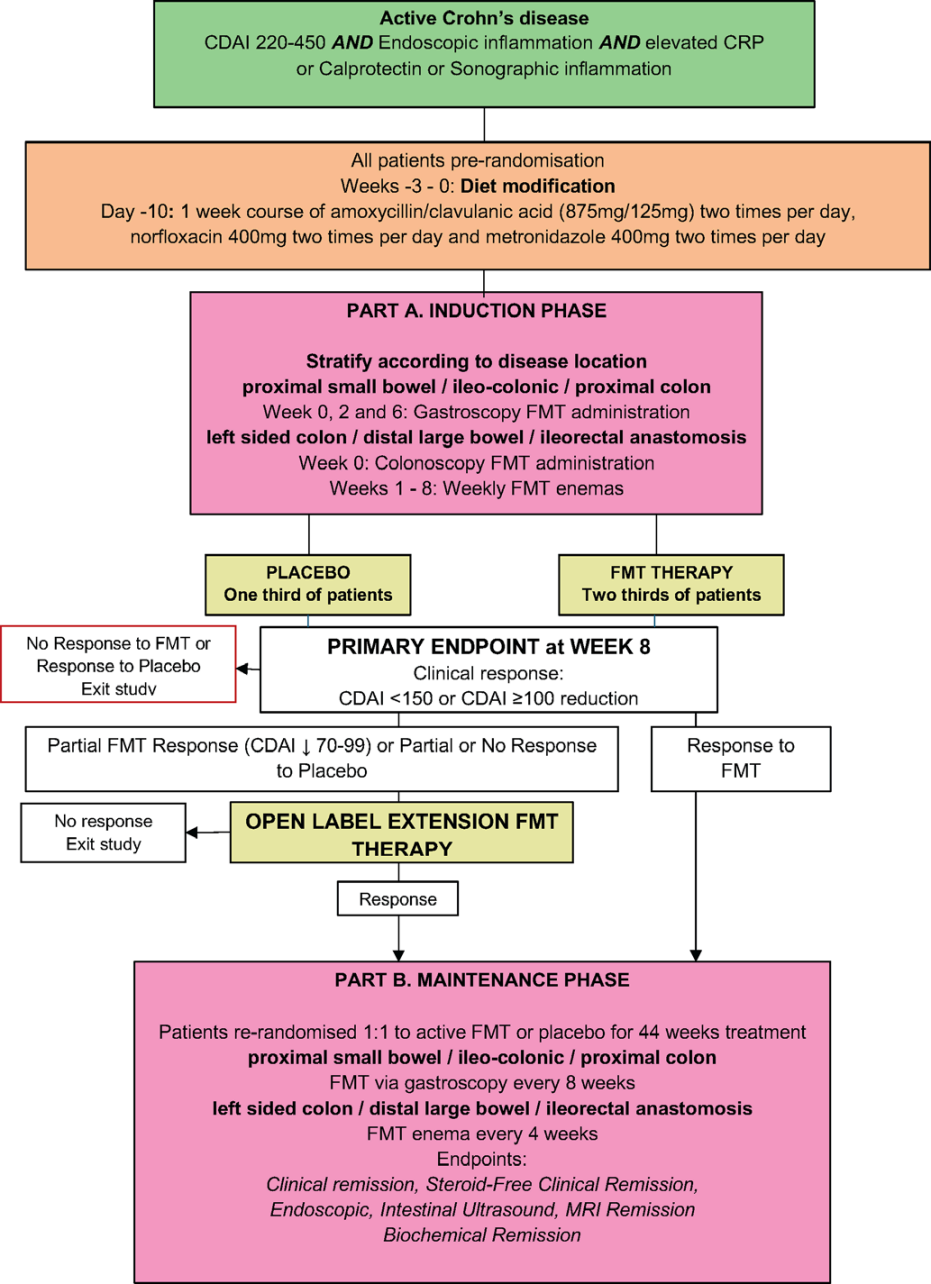
Microbial Restoration study design. CDAI, Crohn’s Disease Activity Index; CRP, C reactive protein; FMT, faecal microbiota transplantation.

The study design is detailed in figure 1.

### Optimisation phase

Prior to randomisation, both study arms will be prescribed a course of antibiotic therapy, in addition to recommended dietary modification.

#### Antibiotic administration

All patients receive at day −10, for 7 days, a course of antibiotics comprising amoxycillin with clavulanic acid, metronidazole and norfloxacin (dosages: 1 g per oral two times per day; 400 mg per oral two times per day; and 400 mg per oral two times per day, respectively). Antibiotics cease 3 days prior to FMT or placebo administration.

#### Dietary modification

All patients will see a dietitian at study entry. Patients will be provided with detailed information on dietary modification to be commenced at study entry, 3 weeks prior to FMT or placebo administration, and continued throughout the study. The diet is a plant-enriched, whole-food approach that is free of food additives. Written dietary recommendations and suggested meal options and recipes are included on the MIRO study phone app.

### Induction phase

Patients assigned to receive upper gastrointestinal delivery of FMT will undergo endoscopic administration of FMT or placebo into the distal duodenum or jejunum at weeks 0, 2 and 6.

Patients receiving lower gastrointestinal delivery will undergo endoscopic administration of FMT or placebo into the ileum or right colon at week 0, followed by weekly FMT or placebo enemas until week 8.

At the week 8 assessment table 2, patients achieving a clinical response to FMT (CDAI decrease of ≥100 points or CDAI <150) enter the maintenance phase. Patients who achieve a partial response to FMT (CDAI decrease of <100 and >70 points) will be offered open-label extension of FMT for a further 8 weeks; if they fail to achieve complete clinical response to FMT at week 16, they will exit the study. Patients receiving initial (induction) placebo will be offered 8 weeks of open-label induction active FMT therapy; if they fail to achieve complete clinical response to FMT at week 16, they will exit the study. Patients receiving placebo therapy who achieve a clinical response at week 8 will exit the study.

**Table 2:** Clinical outcomes assessments

	Method of assessment	Timepoints
Disease activity	Crohn’s Disease Activity Index	Weeks 0, 2, 6, 8 (±16), 16 (or 24), 24 (or 32), 32 (or 40), 40 (or 48), 48 (or 56), 52 (or 60)
Biomarkers	Blood: C-reactive protein	Weeks 0, 2, 6, 8 (±16), 16 (or 24), 24 (or 32), 32 (or 40), 40 (or 48), 48 (or 56), 52 (or 60)
	Stool: calprotectin	Weeks 0, 2, 8 (±16), 24 (or 32), 52 (or 60)
Sonographic assessment	IUS BWT <3 mm and/or Limberg Score 0 or 1; MRI BWT <4 mm and no/minimal wall enhancement	Weeks −3, 8 (±16), 52 (or 60)
Quality of life measures	Short-IBD-Q and EuroQol (EQ5D)	Weeks −3, 8 (±16), 52 (or 60)
Psychological assessment	Hospital Anxiety and Depression Scale (HADS)	Weeks −3, 8 (±16), 52 (or 60)
Endoscopy assessment	SES-CD	Weeks 0, 8 (±16), 52 (or 60)
Histological assessment	Presence of acute inflammation; Robarts Histology Index	Weeks 0, 8 (±16), 52 (or 60)
Diet tolerability	24-hour diet recall; Diet compliance assessment; Food additive survey	Weeks −3, 0, 8 (±16), 52 (or 60)
Safety	Surveillance	Duration of trial and up to 5 years following withdrawal/exit

BWT, bowel wall thickness; SES-CD, simplified endoscopic activity score for Crohn's disease.

### Open-label phase

The open-label therapy is administered according to the same schedule assigned to the patients in the induction phase. After 8 weeks of open-label FMT, patients will have another disease activity assessment; patients achieving a clinical response (CDAI decrease of ≥100 points or CDAI <150) from screening to end week 16 will enter the maintenance study.

### Maintenance phase

Patients who have had a clinical response at week 8 (or week 16 for those who received open-label FMT extension) will enter the maintenance study and will be re-randomised 1:1 to receive either active FMT or placebo for the remaining 44 weeks. Patients receiving upper gastrointestinal delivery will undergo endoscopic administration of FMT into the duodenum or jejunum every 8 weeks for 44 weeks. Patients receiving lower gastrointestinal delivery will undergo FMT enemas every 4 weeks for 44 weeks. At the week 52 assessment, or week 60 for those who received open-label FMT extension, the patients and study team will remain blinded.

Biological specimens will be collected in line with the study protocol and standard study procedures.

### Interventions

#### Treatment arm

##### Infusion product

The FMT product comprises whole stool prepared anaerobically and frozen. The frozen product will be thawed at room temperature for 4 hours prior to administration.

The administered FMT comprises 25% faecal material by weight, 65% normal saline and 10% glycerol.

##### FMT dose

FMT dose is 12.5 g of stool per 50 mL syringe.

Upper gastrointestinal delivery group: endoscopic administration of (25 g) 100 mL of FMT or placebo at each procedure (total induction dose: 75 g; total maintenance dose: 125 g).

Lower gastrointestinal delivery group: endoscopic administration of (50 g) 200 mL of FMT, followed by 100 mL of FMT or placebo enemas (total induction dose: 225 g; total maintenance dose: 250 g).

### Control arm

#### Placebo product

The placebo product contains 0.05% brown dye (combination mixture of HT Brown and Brilliant Blue FCF) in 0.9% normal saline and 10% glycerol. The product has been developed to be identical in appearance to FMT. Odourant is not used in this study as it is not able to be used according to Australian regulatory TGA guidelines.

### Preservation of double blinding

The participants, clinicians, research team and outcome assessors will be blinded to treatment allocation. The placebo product, packaging and labelling will be identical in appearance to the FMT product. The barcode numbers are indistinguishable between placebo and FMT products. The placebo product is frozen, stored and handled identically to the FMT product at each hospital site to prevent unblinding of study investigators during retrieval.

Unblinding will take place the day prior to the week 8 assessment of the induction phase to allow preparation of FMT or placebo for the open-label phase as FMT or placebo endoscopic infusion requires 4 hours of thawing. This unblinding will be undertaken by a designated team member who is not involved in the clinical running of the trial; they will allocate the maintenance syringe kit number. The study assessment, including IUS and endoscopy, will be performed by a study member who remains blinded to the treatment arm. In the event that a patient needs to be unblinded for medical care, a 24-hour contact number is available for immediate unblinding.

### Additional therapies

#### Metoclopramide

Intravenous metoclopramide 10 mg will be administered at the time of sedation by the anaesthetist to reduce the risk of aspiration through its antiemetic and prokinetic properties.

#### Loperamide

For patients with an ileorectal or ileosigmoid anastomosis, a dose of 4 mg or 2 mg of loperamide will be administered an hour prior to FMT administration via colonoscopy or enema, respectively, to maximise exposure of the FMT prior to defecation post-FMT.

#### Concomitant Crohn’s disease therapies

Patients will continue stable doses of their prestudy Crohn’s disease therapies, except for rectal therapies, which are not permitted due to the risk of trauma and potential subsequent bacteria translocation. Stable doses for 2 months prior to the study are required for immunomodulators and biologic agents, 1 month for small molecule inhibitors and 2 weeks for aminosalicylates.

#### Corticosteroid use

Participants can enter the study on prednisolone 20 mg or below, or budesonide 6 mg or below. However, mandatory corticosteroid tapering will be required from week 0 of 2.5 mg of prednisolone per week or 3 mg of budesonide every 2 weeks to ensure that participants do not remain on corticosteroids at the primary endpoint assessment.

Commencement of corticosteroid therapy after study entry will be considered a protocol deviation. However, patients can remain in the study unless it occurs within 4 weeks of the primary endpoint assessment, or if greater than two courses of more than 1 month duration are required during the entire study, either of which would be considered a protocol violation warranting withdrawal.

#### Antibiotic use

The use of antibiotic therapy greater than two courses lasting more than 1 week within a year will be considered a protocol violation warranting withdrawal. Topical antibiotics are permitted.

### Early discontinuation/withdrawal/treatment failure

Patients may be withdrawn from the study if any of the following occurs:

- The patient requests withdrawal from the study.
- Fails to attend one of the major study visits.
- Treatment failure, defined as:
  - Clinical recurrence: symptoms typical of Crohn’s disease, necessitating unscheduled endoscopic or surgical intervention or addition of new drug therapy, associated with confirmed active disease.
  - Failed corticosteroid withdrawal.
  - Serious adverse events or complications necessitating cessation of therapy.
- Pregnancy.
- The investigator can also decide to withdraw a patient from the study if he/she considers it as necessary (eg, subsequent non-respect of inclusion criteria, a change in diagnosis).

At the time of withdrawal/discontinuation, patients will undergo a disease activity assessment including CRP, calprotectin, sonographic and endoscopic assessment if possible.

### MIRO study phone app

The MIRO app (developed by KeyLead Health) will be used to collect all patient-reported outcomes, from which they will be automatically integrated into the clinical database. This app is customised to track and process participants’ trial-related activities in real time. Patient’s confidentiality is protected by adherence to the Australian Privacy Principles established by the Privacy Act 1988. The data platform has Protected Certification from The Australian Signals Directorate and is Health Insurance Portability and Accountability Act compliant. Data are encrypted using the Advanced Encryption Standard with a 256-bit key standard at rest and with Transport Layer Security/Secure Sockets Layer at transit.

### Data monitoring

The audit plan will involve 100% manual verification checks on a 10% random sample of participant files.

### Safety monitoring for patients

Medication or procedure-related adverse events will be monitored during the procedures and at each subsequent visit, with details of the event recorded on the electronic case report form using standard terminology as per the Common Terminology for Adverse Events (V .5.0). A safety monitoring committee will review all cases and adverse events which occur within the context of this cohort. The safety monitoring committee will convene at least weekly to review all patients’ safety and at the time of an adverse event.

### Patient involvement

Patients were not involved in the design and conduct of this research.

### Statistical analysis

Primary analysis will be using modified intention-to-treat population, that is all patients who were randomised and received at least one dose of the study treatment. Per-protocol analysis will be performed as a secondary analysis.

The primary endpoint is the achievement of clinical response at week 8, defined as either a reduction in CDAI of ≥100 points or a CDAI score of <150 points. This primary outcome will be analysed using a general linear model with binomial distribution and log link, with results presented as risk ratio and 95% CIs. A two-sided p value of less than 0.05 will be considered statistically significant.

All binary secondary outcomes in both the induction and maintenance phases will be compared between arms using a general linear model with binomial distribution and log link. Time taken to achieve a clinical outcome will be compared between groups using Kaplan-Meier survival analysis and Cox regression model.

All continuous secondary outcomes will be compared between groups using analysis of covariance. Model fit will be assessed by visual inspection of the residuals, and outcomes might be transformed if required. If the transformation will not yield improved model fit, then the rank sum test will be used for comparison.

There will not be corrections for multiple comparisons, but the results will be interpreted in view of multiple comparisons.

The number of participants in the open-label phase extension is expected to be small, providing for mainly descriptive analysis. However, to gain a larger impression of the impact of active FMT versus placebo, combined active blinded and unblinded FMT outcomes compared with placebo will be assessed.

The primary aim of the open-label induction phase is to enable the majority of participants in the study to access FMT therapy.

Subgroup analyses will include outcomes in the upper and lower gut delivery cohorts, as well as many of the patient demographic variables.

No interim analyses will be performed. The missing data strategy will depend on the type of intercurrent event.

### Ethics and dissemination

#### Ethics

This clinical trial will be conducted in compliance with all stipulations of this protocol (version 1.4), the conditions of the St Vincent’s Hospital Melbourne Human Research Ethics Committee approval, the National Health and Medical Research Council (NHMRC) National Statement on ethical Conduct in Human Research (2007 and all updates), the Integrated Addendum to ICH E6 (R1): Guideline for Good Clinical Practice E6 (R2), dated 9 November 2016 annotated with TGA comments and the NHMRC guidance safety monitoring and reporting in clinical trials involving therapeutic goods (EH59, 2016).

#### Data sharing

The principal investigators will be jointly responsible for the dissemination of results arising from this project. Results will be disseminated by presentation at medical conferences and publications in peer-reviewed medical journals. Data can be accessed by request to the principal investigator.

## Discussion

Despite the recognised central role of the microbiome in Crohn’s disease pathogenesis, no large-scale placebo-controlled trials have been conducted to date examining FMT for this indication. FMT is an established treatment option for patients with recurrent *Clostridioides difficile* infection, and more recently as an effective therapy for the induction and maintenance of remission in patients with UC. Many FMT studies have been performed for other gastrointestinal indications; however, no universal guidelines exist to direct use of FMT with regard to mode of administration, dose or treatment intervals.

The MIRO study protocol has been designed to best examine the efficacy of FMT in Crohn’s disease.

The sample size calculation is based on an alpha value of 0.05 (two sided), power of 80%, an expected rate of the primary outcome of 60% in the intervention arm and 30% in the comparator arm. Allowing for a dropout rate of 20%, a total of 120 patients will be required to achieve a non-dropout number of 96 patients completing the induction phase of the study.

The allocation of donors to patients will be at least eight patients for each donor, to enable the identification of donor effects. An anaerobic processing technique will be used to best preserve obligate anaerobes, hypothesised to be key organisms in efficacy,^27^ given their reduced abundance in cohorts of patients with Crohn’s disease.^1^ The stool preparation will be in liquid form rather than encapsulated lyophilised stool to maximise the viability of bacterial species. Freezing FMT for storage at −80°C is a practical necessity to enable participants to receive a single donor for the duration of the trial, for transportation between sites, for the screening process of donor stool at a TGA approved Good Manufacturing Practice facility and to allow for the required 3-month quarantine phase prior to retesting. The FMT will be frozen with 10% glycerol at −80°C and thawed for administration. Culture-based viability studies have previously demonstrated minimal impacts on different bacterial groups when freezing faecal material at −80°C.^26^

The MIRO study incorporates two additional microbiota-modulating interventions, making the optimisation phase a unique element of the study design. The dietary advice, administered by study dieticians, incorporates a predominantly whole-food diet that limits processed foods, an established strategy in the management of Crohn’s disease in line with recently published The International Organization for the Study of Inflammatory Bowel Diseases guidelines.^31^,^35^ Recent studies exclude emulsifiers and food additives in patients with Crohn’s disease due to their negative impact on microbial profiles.^36 37^ We included a compliance assessment at the end of the induction and maintenance phases. This will allow us to assess the impact of dietary intervention adherence on outcomes.

The rationale for pre-FMT antibiotic use is twofold: (1) to reduce luminal bacterial load, thereby improving donor microbial engraftment^38^,^40^ and (2) targeting *Proteus mirabilis* species, given its recently recognised pathogenic qualities in Crohn’s disease.^37^ Animal and human studies have demonstrated improved donor engraftment when using combination broad-spectrum antibiotic therapy.^38^,^41^

Pre-FMT or placebo microbial manipulation with dietary modification and antibiotic therapy are applied to all study patients prior to randomisation, making the only variable between groups FMT or placebo administration.

The mode of administration reflects the intention for the therapy to be applied directly to the site of disease. Stratification according to disease extent will therefore determine the route of administration to allow disease surface coverage. Gastroscopic delivery is the modality of choice for small bowel disease. Colonoscopic delivery for proximal colonic disease would be a direct approach; however, repeated colonoscopies are not practical and also have the potential to disturb the microbiota with bowel preparation. Therefore, patients with proximal colonic disease will also receive gastroscopic administration. Patients with left-sided disease or those who have an ileorectal or ileosigmoid anastomosis will receive a colonoscopic infusion followed by weekly enemas. This route was effective in FMT studies in UC.^9 18^

Other practical considerations regarding the route of administration include tolerability and safety. There will be several protocolised measures to mitigate the rare but significant risk of aspiration pneumonia with gastroscopic delivery, including performing the procedure with the patient reclining at 45°, the use of a paediatric colonoscope for administration directly into the distal duodenum/jejunum, low FMT volume (100 mL), intraprocedural intravenous metoclopramide and postprocedure recovery in the upright position.

The FMT doses are based on prior FMT in UC studies,^9 18^ as well as the two randomised controlled trials conducted in Crohn’s disease to date.^23 24^ Additionally, systematic reviews evaluating the dose of FMT in *Clostridioides difficile* infection revealed that multiple FMT is more effective than single FMT and that the reduced efficacy of a dose<50 g of stool has been compensated for with the increased cumulative dose of multiple administrations.^42 43^

The use of a clinical score for entry and as the primary endpoint is consistent with the current clinical trial standards for Crohn’s disease, according to US Food and Drug Administration clinical trials guidelines (ref website: https://www.fda.gov/regulatory-information/search-fda-guidance-documents/crohns-disease-developing-drugs-treatment). However, the lack of objectivity of this endpoint makes it challenging to interpret in certain settings. Extensive additional clinical, biochemical, sonographic and endoscopic data will be collected to provide objective assessment of disease activity, reflecting the recent shift towards objective assessment of therapeutic interventions.

For ethical reasons, an open-label extension allows initial placebo-treated patients to access FMT, and patients’ Crohn’s disease medications are continued at a stable dose.

An open-label FMT induction extension requires unblinding of patients at week eight to determine the next phase of trial progression. To maintain blinding throughout the maintenance phase of the trial, patients are re-randomised on entry into this phase.

Studies evaluating FMT in UC have demonstrated associations between specific organisms in donor FMT and the extent of engraftment with therapeutic response.^9 18 44^ A limitation of the current scientific literature is the variability in culturing and sequencing techniques and depth of sequencing capabilities. A recent advancement in combining genome and culture techniques has facilitated enhanced metagenomic sequencing, improving taxonomic classification down to subspecies level.^45 46^ This study will use this technology to help establish the mechanism of action of FMT in Crohn’s disease and define the microbial and associated metabolic factors central to Crohn’s disease pathogenesis and treatment response. Identifying specific bacterial factors associated with FMT therapeutic benefit and failure will lead to a more sophisticated approach to the development of microbial therapeutics in IBD and a more personalised approach to treating Crohn’s disease.

The MIRO study will define the therapeutic value of FMT in Crohn’s disease. The study design, a large randomised double-blind placebo-controlled trial, aims to definitively establish this proof of concept.

## Supplementary material

10.1136/bmjopen-2024-094714 online supplemental file 1

10.1136/bmjopen-2024-094714 online supplemental file 2
