# Supplementary material for: Faecal microbiota transplantation in Crohn’s disease: an Australian randomised placebo-controlled trial protocol
Source: BMJ Open. 2025 Apr 19;15(4):e094714. doi: 10.1136/bmjopen-2024-094714 (PMC12010309; doi:10.1136/bmjopen-2024-094714)
Supplement: online supplemental file 2 [file bmjopen-15-4-s002.docx]

**Participant Information Sheet/Consent Form**

**Interventional Study** - *Adult providing own consent*

***[Site Name]***

| **Title** | Faecal Microbiota Transplantation (FMT) In Crohn’s Disease: The MIRO II (Microbial Restoration) Study |
| --- | --- |
| **Short Title** | The MIRO II Study |
| **Project Sponsor** | St Vincent’s Public Hospital, Melbourne |
| **Coordinating Principal Investigator/ Principal Investigator** | Professor Michael Kamm |
| **Location** |  |

**Part 1 What does my participation involve?**

**1 Introduction**

You are invited to take part in this research project. This is because you have Crohn’s Disease. The research project is testing a new treatment for active Crohn’s Disease. The new treatment is called Faecal Microbial Transplantation (FMT).

This Participant Information Sheet/Consent Form tells you about the research project. It explains the tests and treatments involved. Knowing what is involved will help you decide if you want to take part in the research.

Please read this information carefully. Ask questions about anything that you don’t understand or want to know more about. Before deciding whether or not to take part, you might want to talk about it with a relative, friend or your local doctor.

Participation in this research is voluntary. If you don’t wish to take part, you don’t have to. You will receive the best possible care whether or not you take part.

If you decide you want to take part in the research project, you will be asked to sign the consent section. By signing it you are telling us that you:

• Understand what you have read

• Consent to take part in the research project

• Consent to have the tests and treatments that are described

• Consent to the use of your personal and health information as described.

You will be given a copy of this Participant Information and Consent Form to keep.

**2 What is the purpose of this research?**

Inflammatory bowel diseases such as Crohn’s Disease are chronic, disabling conditions affecting the bowel. They develop when there is an interaction between your immune system and the bacteria in your bowel. Whilst there are many different medications that can be used to treat Crohn’s Disease (including steroids, immunosuppressant and biological drugs), these medications are not effective for all patients. There is no known cure for Crohn’s Disease.

FMT is approved in Australia and overseas to treat recurrent or resistant *Clostridium difficile* infection. This is a common infection that occurs due to an imbalance of gut bacteria, which the FMT corrects. The first line treatment for this infection is antibiotics, which often doesn’t completely cure the infection as it comes back. FMT cures the condition in more than 90% of patients.

However, FMT it is not approved in Australia to treat Crohn’s Disease. Small studies have shown FMT to be beneficial in the treatment of another type of inflammatory bowel disease called Ulcerative Colitis even when it is not responding to standard medical therapies. It is not yet known if FMT is a useful treatment for Crohn’s disease.

The MIRO study will define the value of Faecal Microbiota Transplant (the transplanting of someone's faeces into someone else for medical benefit) in Crohn’s disease and its mechanism of action. This study is being done to investigate if FMT can get your active disease under control (in remission) and if it can help you maintain this state of remission. Therefore, it is an experimental treatment for Crohn’s disease and must be tested to see if it is an effective treatment. It has the potential to change the Crohn’s disease therapeutic landscape.

For this study, faeces will be donated from healthy volunteers who have been comprehensively tested for infections and diseases to ensure that these are not passed on in the FMT. Patients in this study will only one donor for the duration of the trial. The faeces will be donated, and the FMT prepared, at a laboratory that meets strict quality and monitoring standards. The preparation process will attempt to limit modification to the faeces in order to give as close to ‘whole stool’ as possible. A preservative and salty water will be combined with the faeces for storage purposes.

Results from this study will contribute to the understanding of microbial therapies in the treatment of inflammatory bowel diseases such as Crohn’s Disease and will contribute to the future development and accessibility of these types of treatments for patients in the future.

This study is has been funded by the National Health and Medical Research Council (NHMRC), the Australian Gastro Intestinal Research Foundation and a grant from The Leona M. and Harry B. Helmsley Charitable Trust.

**3 What does participation in this research involve?**

You will be screened for eligibility in the study and if meeting eligibility criteria will be required to read and sign this consent form prior to any assessments, procedures or treatment being performed.

To be eligible for this study, you will need to be over the age of 18 and have symptoms of active Crohn’s disease, and have evidence of disease activity on colonoscopy. If this colonoscopy has not been performed within the six months prior to study enrolment it will need to be performed before you can receive treatment.

You will be participating in a randomised controlled research project. Sometimes we do not know which treatment is best for treating a condition. To find out we need to compare different treatments. We put people into groups and give each group a different treatment. The results are compared to see if one is better. To try to make sure the groups are the same, each participant is put into a group by chance (random). To participate in this study you will first be randomly assigned (by chance) to one of two groups, FMT or placebo. You will have a two in three (66%) chance of receiving the FMT treatment. A placebo is a medication with no active ingredients. It looks like the real thing but is not.

You will be participating in a double-blind study. This means that neither you nor your study doctor will know which treatment you are receiving. However, in special circumstances your study doctor can find out which treatment you are receiving.

As a study participant, you will need to undergo a screening process prior to FMT or placebo treatment. You will need to attend an appointment with one of the study doctors who will ask a series of questions. You will need to have a blood test and faecal tests performed. These tests will screen for major health conditions (including liver or kidney problems), and infection and inflammation. Screening tests will be performed for infections including HIV, viral hepatitis and tuberculosis. The screening process is comprehensive, for your safety.

Faecal material for transplantation will be collected from a suitable, healthy donor who has undergone extensive screening for transmissible infections. The FMT is being purchased from BiomeBank, a government approved private laboratory located in Adelaide that collects and produces faecal transplants. The faecal material will be prepared in a laboratory in a solution with normal saline (salty water) and glycerol to protect the transplant for freezing.

The FMT will intially be given by either a gastroscopy or colonoscopy depending on the location of your disease. A gastroscopy is a test in which a lighted tube is passed through your mouth to look at your stomach and the top part of your small intestines. A colonoscopy is a test in which a lighted tube is passed through the rectum into the entire bowel (colon and part of the small bowel). You will likely have previously had one of these procedures to diagnose and monitor your Crohn’s disease. Which procedure you need will be determined by which parts of your bowel are most affected by your Crohn’s Disease. This will be determined by the study doctor in your initial visit. Your endoscopies will be performed by an experienced gastroenterologist under procedural sedation.

Some questionnaires will be performed by your doctor or a member of the study team. We will also ask you to download a smart phone application called “MIRO” that will periodically alert you and ask you to provide information on your condition, and medications while you are in the study. If you do not have a smart phone, other methods of collecting this information can be used instead.

All participants prior to their treatment will have a consultation with our study dietitian who will recommend you modify your normal diet, as a food additive free diet has been shown to assist with treating Crohn’s disease. We think that the foods you eat may help reduce inflammation in combination with the FMT, and we will support you to make changes to your diet as needed. The cost of food should be no more than what you normally pay for food. We will be assessing how well the diet was followed on a few occasions during the study (weeks 0, 8 and 52) so that we can compare how well the FMT worked between participants who followed the diet and those who did not. There will be no consequences for patients who do not follow the diet. These patients will not be removed from the trial.

Ten days prior to starting treatment you will be required to take a 7-day course of three different antibiotics to create the best environment in your bowel for the FMT to be effective. If you are allergic or intolerant to any of these antibiotics you will not be required to take that medication and will be able continue to participate in the study.

This study has three phases (see Figure 1) –

1. Induction (getting active disease under control) – all participants will take part for the FIRST 8 weeks.
2. Open label – not all participants will take part in this phase (8 weeks).
3. Maintenance (keeping disease activity under control) – only participants that have had some benefit from this treatment will continue in this part of the study (44 weeks).


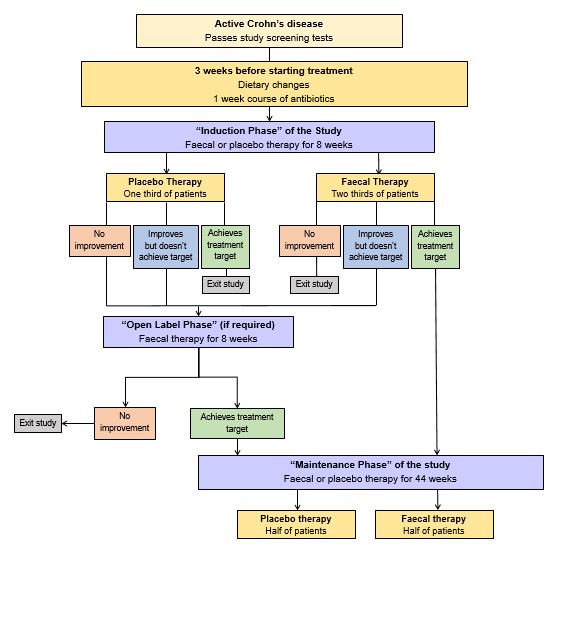


***Figure 1. Study Design***

For the **INDUCTION** phase:

After you have been screened to ensure you are suitable for the study, you will be randomly allocated to a treatment group (active FMT or Placebo, two in three patients will initially receive FMT), and we will commence your treatment.

The location of your Crohn’s disease will determine how your treatment will be administered. If you have:

1. Upper gastrointestinal Crohn’s Disease (affecting the top part of your gut, the stomach, small intestine or upper part of your colon) – FMT or placebo will be administered via Gastroscopy at study commencement, week 2 and week 6.

OR

1. Large bowel Crohn’s Disease (affecting the bottom part of your bowel, your lower colon) – FMT or placebo will be initially administered by colonoscopy at study commencement and then home enemas administered by yourself, once a week for 7 weeks..

After 8 weeks, all participants will be assessed by endoscopy (colonoscopy and/or gastroscopy), imaging (MRI or Ultrasound) and asked a series of questions about your symptoms and your quality of life.

Blood tests, faecal tests and questionaries will be required during your treatment. Blood tests will be performed to check for inflammation as well as check your overall health. Faecal testing will be performed to detect any infection and to quantify inflammation in the bowel. During this phase you will have 4 study visits (including your FMT administrations if by gastroscopy and week 8 assessment) that will either be in person or via phone depending on which group you are in. On 3 of these occasions you will have a blood and faecal sample taken.

After this assessment, the study team will disclose to you which treatment you received, active FMT or placebo.

*If you were assigned* ***active FMT treatment for the INDUCTION Phase*** *(first 8 weeks of the study):*

We will then assess if you have had any benefit from the first course of treatment. If you have met the target response at this time, we will again randomise you to placebo or active FMT treatment, but you will have a lower chance of receiving active treatment (one in two patients will receive FMT, or 50%). You will then continue your treatment for an additional 44 weeks (for a total period of 52 weeks; 12 months), this is called the “maintenance phase” of the study.

If you have not had any response at 8 weeks, you will be withdrawn from the study.

If you have improved on active treatment but did not meet the target response we will continue your treatment for an additional 8 weeks, the “open label phase” of the study. You will know that you are receiving active FMT treatment, no patients will receive placebo during this phase.

The administration of the FMT will be the same as the first 8 weeks, either by gastroscopies or a colonoscopy + home enemas, depending on where your disease is located.

At the end of this “open label phase” (16 weeks) we will again assess your response by endoscopy (colonoscopy and/or gastroscopy), imaging (MRI or Ultrasound) and ask a series of questions about your symptoms and your quality of life.

If you have met the target response at this time, we will again randomise you to placebo or active FMT treatment, but you will have a lower chance of receiving active treatment (one in two patients will receive FMT, or 50%). You will then continue your treatment for an additional 44 weeks (for a total period of 60 weeks; 15 months), this is called the “maintenance phase” of the study.

*If you were assigned* ***placebo for the INDUCTION Phase*** *(first 8 weeks of the study):*

If you have met the response target while on placebo treatment, we will withdraw you from the study and you will not receive further treatment.

We would expect you not to have responded to placebo in the first 8 weeks, and you will therefore now be able to access active FMT for a further 8 weeks, this is called the “open label phase” of the study. You will know that you are receiving active FMT treatment, no patients will receive placebo during this phase.

The administration of the FMT will be the same as the first 8 weeks, either by gastroscopies or a colonoscopy + home enemas, depending on where your disease is located.

At the end of this phase (16 weeks) we will again assess your response by endoscopy (colonoscopy and/or gastroscopy), imaging (MRI or Ultrasound) and ask a series of questions about your symptoms and your quality of life.

If you have met the response target at this time, we will again randomise you to placebo or active FMT treatment, but you will have a lower chance of receiving active treatment (one in two patients will receive FMT, or 50%). You will then continue your treatment for an additional 44 weeks (for a total period of 60 weeks; 15 months), this is called the “maintenance phase” of the study.

For the **MAINTENANCE** phase:

After 8 weeks if you have responded to the initial course of FMT (or you have responded to a further 8-week Open label FMT treatment) you will be randomised to FMT or placebo once more with one in two patients receiving FMT to continue onto the Maintenance arm of FMT administration.

If you have:

1. Upper gastrointestinal Crohn’s Disease (affecting the top part of your gut, the stomach, small intestine or upper part of your colon) – FMT or placebo will be administered via Gastroscopy at every 8 weeks for 44 weeks (5 gastroscopies).

OR

1. Large bowel Crohn’s Disease (affecting the bottom part of your bowel, your lower colon) – FMT or placebo will administered by home enemas every 4 weeks for 44 weeks (11 enemas).

Following this you will have your Crohn’s disease assessed once more at the end of the study via endoscopy, imaging (MRI or Ultrasound) and symptom and quality of life questionnaires.

During this phase you will have 6 study visits at most every 2 months (including your FMT administrations if by gastroscopy and end of study assessment) that will either be in person or via phone depending on which group you are in. On all of these occasions you will have a blood sample taken. On 2 of these visits a faecal sample will be required.

Follow up study visits will also be required throughout the study following FMT to assess your response to treatment. It is important that you attend all study visits.

The total study period will be 52 or 60 weeks – depending on your response to the induction therapy.

If you are in the group requiring home enema administration you will be required to come in for an education session for the administration of your first enema. Following this, each enema will need to be picked up on the day of planned administration as they cannot be stored in home freezers for longer than one day to maintain the product. The enema frequency is weekly for induction period (6 enema pick ups) and every 4 weeks for the maintenance phase (11 pick ups).

There are no additional costs associated with participating in this research project, nor will you be paid. All medication, tests and medical care required as part of the research project will be provided to you free of charge. Participants will not be reimbursed for time, travel or parking.

Although there are no established long-term safety concerns around the use of FMT, you are required to participate in a long-term safety database if you receive a faecal transplant, this will provide long term information on this treatment. We may contact you by phone from time to time to obtain long term information on your health that may be related to your faecal transplant and your inflammatory bowel diseases, but this will not require any in person visits or medical tests. The database will be managed by the Department of Gastroenterology under strict security conditions.

You will be required to provide ongoing health information into the surveillance database for up to 10 years.

This research project has been designed to make sure the researchers interpret the results in a fair and appropriate way and avoids study doctors or participants jumping to conclusions.

If you decide to participate in this research project, the study doctor will inform your local doctor.

If your study doctor decides your treatment is not working, or if your medical condition deteriorates or if there is new information about your medical condition that makes it no longer appropriate for you to continue, you may be withdrawn from the study.

**4 What do I have to do?**

To participate in this study, we require you to attend scheduled appointments, endoscopy procedures, provide test samples and complete questionnaires over a 13-15 month period, and follow up with us for up to 10 years. You will be required to attend an initial appointment with a study doctor to determine eligibility and to arrange for the screening blood and stool testing. As part of this appointment you will need to have your weight and height measured, and we will also ask about your health and travel history, and any drugs or medications you may take.

You may need to make dietary changes throughout the study and this will be discussed on your initial visit with the study dietitian, who will be available for the duration of the study if you have any questions. Throughout the study you are still able to engage in physical activity and participate in sports if you wish.

In most cases you will still be able to take your regular medications during the study period. It is important that you advise study doctors of all the medications and over the counter preparations that you are taking at study entry.

If you wish to start a new medication for any reason during the trial this must be discussed with study doctors first. There may be some restrictions on commencing particular medications during the trial including steroid medications (such as prednisolone or budesonide), new Crohn’s drug therapy, reflux medication, over the counter supplements including probiotics, and any antibiotics for any reason.

You are not able to donate blood while you are enrolled in this study.

If you are pregnant or intend on becoming pregnant within 12 months of entering the trial you are not able to take part in this study.

Patients from regional centres will not be excluded but will be counselled on the need to attend for all required in person visits.

By consenting to take part in this study you agree to receive FMT administered by doctors at St Vincent’s Hospital or Queen Elizabeth Hospital and agree to attend all study visits and procedures in accordance with the instructions provided. You will also be contacted by phone by a member of the study team to collect long term data on your health for a long-term database of recipient outcomes.

**5 Other relevant information about the research project**

This is a multi-centre trial that will involve patients from the major Inflammatory Bowel Disease (IBD) centres in Melbourne and the Queen Elizabeth Hospital in Adelaide, South Australia. Patients will be looked after by the team at St. Vincent’s Hospital or Queen Elizabeth Hospital for the duration of the study with regular communication provided to your regular gastroenterologist.

We aim to enrol 120 participants into this study, with 60 patients to be enrolled at each study site.

**6 Do I have to take part in this research project?**

Participation in any research project is voluntary. If you do not wish to take part, you do not have to. If you decide to take part and later change your mind, you are free to withdraw from the project at any stage.

If you do decide to take part, you will be given this Participant Information and Consent Form to sign and you will be given a copy to keep.

Your decision whether to take part or not to take part, or to take part and then withdraw, will not affect your routine treatment, your relationship with those treating you or your relationship with St Vincent’s Hospital.

**7 What are the alternatives to participation?**

You do not have to take part in this research project to receive treatment at this hospital. Other options are available for Crohn’s Disease; these include steroid medications, immunomodulators (azathioprine, mercaptopurine, methotrexate, tacrolimus), biological drugs (infliximab, adalimumab, vedolizumab, ustekinumab) or surgery. Your study doctor will discuss these options with you before you decide whether or not to take part in this research project. You can also discuss the options with your local doctor.

**8 What are the possible benefits of taking part?**

We cannot guarantee or promise that you will receive any benefits from this research; however, possible benefits may include improvement or resolution of bowel inflammation related to your Crohn’s disease. This may mean that stool frequency reduces, and there is reduction or resolution in rectal bleeding and abdominal pain.

The results of this study may benefit future patients with Crohn’s disease.

**9 What are the possible risks and disadvantages of taking part?**

Medical treatments often cause side effects. You may have none, some or all of the effects listed below, and they may be mild, moderate or severe. If you have any of these side effects, or are worried about them, talk with your study doctor or the research staff. Your study doctor will also be looking out for side effects.

There may be side effects that the researchers do not expect or do not know about and that may be serious. Tell your study doctor immediately about any new or unusual symptoms that you get.

Many side effects go away shortly after treatment ends. However, sometimes side effects can be serious, long lasting or permanent. If a severe side effect or reaction occurs, your study doctor may need to stop your treatment. Your study doctor will discuss the best way of managing any side effects with you.

**Possible Side Effects of Faecal Microbial Transplantation**

There are few established safety concerns around the use of FMT.

Like any treatment for Crohn’s disease, FMT may not work in everyone and rarely, may make symptoms worse. In trials comparing FMT to placebo, no increase in adverse events or side effects from this treatment has been described.

While your faecal donor will undergo extensive screening for infectious diseases, there is a small risk of transmission of a previously un-identified pathogen or drug resistant bacteria, although this is very rare (less than 5 cases world-wide). There is also a small risk of aspiration pneumonia (a lung infection), when FMT is given by gastroscopy, although we take active steps to minimise this risk.

Based on standard clinical practice FMT expiration date is 2 years, which has been approved by the Therapeutics Good Administration. Due to the complexity of the study design, which requires each patient to receive the same donor for the duration of the study, the expiry date used will be 12 months beyond the Therapeutics Good Administration approved date. It important to consider that the disadvantage of prolonged storage is not likely to relate to the safety of the product but rather may reflect reduced effect due to reduced availability of organisms in the product. Biomebank are currently monitoring this with intent to extend the expiry date if, and when, they have this data available.

**Possible Side effects of Antibiotics**

Antibiotics are commonly prescribed medications that are well tolerated by most people. Common mild side effects of the antibiotics used in this study include: nausea (feeling sick on the stomach) or vomiting, diarrhoea, metallic taste in your mouth, muscle pain, headache, rash, and vaginal or oral thrush. Rare side effects include: increased sensitivity to the sun (becoming sunburned more quickly), severe vomiting and/or diarrhoea, flushing of skin. Your study doctor will monitor you for any issues, and we may adjust your medications if you have any side effects.

**Endoscopy (Gastroscopy/Colonoscopy) and Sedation/Anaesthesia**

When FMT is administered via gastroscopy or colonoscopy there are risks associated with these procedures.

A gastroscopy is a test where a lighted tube in passed through the mouth, into the oesophagus and stomach and first part of the small bowel (duodenum). A colonoscopy is a test in which the lighted tube is passed through the rectum into the large bowel (colon) and part of the small bowel. Your physician will give you medication to help you relax or be sedated for either of these procedures. Twelve small biopsies (which are tiny pieces of tissue 1-2mm in size) will be taken from the lining of the stomach and/or bowel for examination when you have a gastroscopy/colonoscopy performed at study commencement, after 8 weeks of induction therapy (and 16 if in open label phase) and after 44 weeks of maintenance therapy. These biopsies will be examined in a laboratory to see if there is active inflammation and to provide information about which bacteria live in the stomach and bowel.

Risks of these procedures may include, but are not limited to: redness and swelling of the veins (called inflammation); excessive sedation if sedation is given; allergic reaction to medication, if medication is given; bleeding of the bowel (gastrointestinal bleeding); and a hole (called perforation) could develop in the bowel from the tube.

| **Adverse Effect** | **How often is it likely to occur?** | **How severe might it be?** | **How long might it last?** |
| --- | --- | --- | --- |
| Bleeding following gastroscopy or colonoscopy | 0.3% (3 in 1000) | Mild | 1-2 days |
| *Abdominal pain following colonoscopy* | 5% (5 in 100) | Mild | 1-2 days |
| Colonic perforation following colonoscopy | Less than 0.1% (less than 1 in 1000) | Severe | Variable |
| *Oesophageal, gastric or duodenal perforation following gastroscopy* | Less than 0.01% (less than 1 in 10000) | Severe | Variable |

Procedural sedation is a type of anaesthesia and will be given to make you comfortable during the colonoscopies performed as part of this study. These days, whilst anaesthesia is generally very safe there are some risks associated with anaesthesia. The most common problems associated with anaesthesia are feeling unwell or vomiting, bruising at the site of injections, sore throat or hoarse voice. Most participants do not have these problems. If these problems do happen, they usually get better very quickly. Damage to teeth may occur, but this is rare. The risk of brain damage or death due to anaesthesia is very rare.

The risk of problems from anaesthesia increases for participants who are having more major surgery, those with medical problems and those that require difficult anaesthetic procedures. If you have any concerns about these issues, you should discuss them with the study team.

**Abdominal Ultrasound****s**

Ultrasound scans use high frequency sound waves to capture live images and video. This helps doctors view the inside of your body. Abdominal ultrasounds are used to visualize the organs and structures (in detail) inside the abdomen. Increasingly, intestinal (bowel) ultrasound has been found to be useful in patients with Crohn’s disease.

An abdominal ultrasound has no risks. Unlike X-rays or CT scans, ultrasounds use no radiation, which is why doctors prefer to use them with pregnant women to examine foetuses as well as in this study to closely monitor the Crohn’s disease strictures. Ultrasound scanning is not painful although you may feel slight discomfort during the procedure. You should let your doctor know right away if the pain becomes severe and the test will be stopped immediately.

**MRI Scans**

MRI stands for magnetic resonance imaging. A MRI scanner is a machine that uses electromagnetic radiation (radio waves) in a strong magnetic field to take clear pictures of the inside of the body. Electromagnetic radiation is not the same as ionising radiation used, for example, in X-rays. The pictures taken by the machine are called MRI scans.

We will ask you to lie on a table inside the MRI scanner. The scanner will record information about your bowel. It is very important that you keep very still during the scanning. When you lie on the table, we will make sure you are in a comfortable position so that you can keep still. The scanner is very noisy and we can give you some earphones to reduce the noise. Some people may experience symptoms of claustrophobia from lying in a confined space. If you do experience discomfort at any time during the scan, you will be able to alert staff by pressing on a call button provided to you.

There are no proven long-term risks related to MRI scans as used in this research project. MRI is considered to be safe when performed at a centre with appropriate procedures. However, the magnetic attraction for some metal objects can pose a safety risk, so it is important that metal objects are not taken into the scanner room.

We will thoroughly examine you to make sure there is no reason for you not to have the scan. You must tell us if you have metal implanted in your body, such as a pacemaker or metal pins.

**Blood Tests**

Blood testing is a simple medical procedure. There may be some mild discomfort or bruising at the site of the blood test which is usually resolves in hours to days and does not require any further treatment.

**Pregnancy**

The effects of FMT on the unborn child and on the newborn baby are not known. For female participants, it is important that you are not pregnant or breast-feeding and do not become pregnant during the course of the research project. You must not participate in the research if you are pregnant or trying to become pregnant, or breast-feeding. If you are female and child-bearing is a possibility, you will be required to undergo a pregnancy test prior to commencing the research project. If you are male, you should not father a child or donate sperm for at least 3 months after the last dose of study medication.

Both male and female participants must avoid pregnancy during the course of the research and for a period of 3 months after completion of the research project. You should discuss effective

methods of avoiding pregnancy with your study doctor.

*For female participants:* If you do become pregnant whilst participating in the research project, you should advise your study doctor immediately. Your study doctor will withdraw you from the research project and advise on further medical attention should this be necessary. You must not continue in the research if you become pregnant.

*For male participants:* You should advise your study doctor if you father a child while participating in the research project. Your study doctor will advise on medical attention for your partner should this be necessary.

**Counselling and Support**

If you become upset or distressed as a result of your participation in the research, the study doctor will be able to arrange for counselling or other appropriate support. Any counselling or support will be provided by qualified staff who are not members of the research project team. This counselling will be provided free of charge.

Should, as part of your participation in this research study, a medical condition of which you were unaware of be uncovered, the study doctors will arrange appropriate medical care. Depending on the nature of the medical condition uncovered it may affect your future participation in this study. Regardless of this our research doctors, clinical scientists study nurses will provide support to you.

**Radiation Exposure**

There will be no radiation exposure as part of this study.

***Should you experience an adverse effect or complication from this treatment you will receive care and management for this at St Vincent’s Public Hospital at no cost as long as you are eligible for Medicare.***

**10 What will happen to my test samples?**

You will be asked to provide consent for the collection of your blood and faeces and tissue during this research project. This is a mandatory component of this research and refusal to provide samples will mean that participants can no longer take part in this study.

The proposed blood tests at the beginning of the study include a screening test for HIV (also called the ‘AIDS’ virus) and Hepatitis. This is because the study doctors need to know if you have HIV or Hepatitis as this may affect your immune system and increase possible side effects of FMT.

Depending on the frequency of COVID-19 (also referred to as ‘Coronavirus’) infections in Australia, you may also need to have a COVID-19 test at various points in the study. The swab taken of the back of your throat and nasal passage will be tested for Covid-19. This is because the study doctors need to know if you might have COVID-19 before coming into the hospital for visits or endoscopies.

You will receive information and counselling before the test. If a test shows you have HIV or Hepatitis, or Covid-19, or any other reportable diseases, you will have follow-up counselling and medical advice. If your test results are positive, the study doctors are required by law to notify government health authorities. Signing the consent form means that you agree to have this testing; it will not be done without your consent.

Your tissue and stool samples will be used to investigate how the microbes in your gut (the “gut microbiome”, which includes bacteria, viruses and fungi) might influence your Crohn’s Disease, and your response to the FMT. This research may assist other Crohn’s Disease patients in the future by identifying reasons why people respond to some treatments and not others. We will also investigate how changes in your diet affect your microbiome, bowel and response to FMT. We will use your blood samples to measure how your immune system responds to the FMT.

Genetic testing specific to Crohn’s Disease may be undertaken at a future date. Genes are made of DNA – the chemical structure carrying your genetic information that determines many human characteristics such as the colour of your eyes or hair. We would like to study your genes in order to understand why some people who have Crohn’s Disease respond to FMT while others do not. Understanding a person’s genes may also be able to explain why some people experience a side effect that others do not. Any information regarding your genes will be de-identified and remain confidential and will not be made publicly available.

Samples of your blood and stool and tissue obtained for the purpose of this research project will be tested and stored at St Vincent’s Hospital Melbourne. The samples will be labelled with an ID number only (not your name) to protect your confidentiality. The information linking your ID number to your name will be kept safely and securely with the research team on password protected computers.

Blood and faecal samples will be taken as part of routine care (for the assessment of inflammation levels and to exclude infection) but will also be stored and tested for research purposes including as above. Your blood tests will be analysed for routine markers of inflammation, liver, kidney and blood function.

We would like to store your blood, stool and tissue specimens for future use in research projects that are an extension of this research project subject to Ethics approval. Your samples may also be included in a sample library that will enable further research related to this project subject to Ethics approval or as a comparison sample in another trial. Some sample testing through a microbial library may enable the development of medications for commercialisation.

Samples of your blood and tissue obtained for the purpose of this research project will be transferred to our scientific collaborators at the University of Queensland, BiomeBank and the Hudson Institute of Medical Research. Your tissue will not be sold by University of Queensland, Biomebank or the Hudson Institute of Medical Research.

Once your blood and/or tissue samples are transferred to the University of Queensland, Biomebank and/or the Hudson Institute of Medical Research, St Vincent’s Hospital (Melbourne) will not be able to control whether the University of Queensland, Biomebank and/or the Hudson Institute of Medical Research transfers or sells your samples at some future date, however St Vincent’s Hospital (Melbourne) will not knowingly transfer your samples to anyone who has expressed intent to sell the samples.

**11 What if new information arises during this research project?**

Sometimes during the course of a research project, new information becomes available about the treatment that is being studied. If this happens, your study doctor will tell you about it and discuss with you whether you want to continue in the research project. If you decide to withdraw, your study doctor will make arrangements for your regular health care to continue. If you decide to continue in the research project you will be asked to sign an updated consent form.

Also, on receiving new information, your study doctor might consider it to be in your best interests to withdraw you from the research project. If this happens, he/ she will explain the reasons and arrange for your regular health care to continue.

**12 Can I have other treatments during this research project?**

Whilst you are participating in this research project, you may not be able to take some or all of the medications or treatments you have been taking for your condition or for other reasons. We will allow most Crohn’s disease therapies if you are on a stable dose but if you require a change in dose or the addition of a new Crohn’s disease therapy you will have to leave the study. You will not be able to enter the study on high doses of steroids or any rectal therapies. If you require steroids during the study we will limit it to two courses that are no longer than four weeks each. If you require antibiotics we will limit it to two courses that are no longer than one week each.

It is important to tell your study doctor and the study staff about any treatments or medications you may be taking, including over-the-counter medications, vitamins or herbal remedies, acupuncture or other alternative treatments. You should also tell your study doctor about any changes to these during your participation in the research project. Your study doctor should also explain to you which treatments or medications need to be stopped for the time you are involved in the research project.

It may also be necessary for you to take medication during or after the research project to address side effects or symptoms that you may have. You may need to pay for these medications and so it is important that you ask your doctor about this possibility.

**13 What if I withdraw from this research project?**

If you decide to withdraw from the project, please notify a member of the research team before you withdraw. This notice will allow that person or the research supervisor to discuss any health risks or special requirements linked to withdrawing.

If you withdraw from this project you may need other types of treatment for your Crohn’s disease. You will be able to discuss this with your research team doctor.

If you do withdraw your consent during the research project, the study doctor and relevant study staff will not collect additional personal information from you, although personal information already collected will be retained to ensure that the results of the research project can be measured properly and to comply with law. You should be aware that data collected by the sponsor up to the time you withdraw will form part of the research project results. If you do not want them to do this, you must tell them before you join the research project.

**14 Could this research project be stopped unexpectedly?**

This research project may be stopped unexpectedly for a variety of reasons. These may include reasons such as:

• Unacceptable side effects

• The drug/treatment/device being shown not to be effective

• The drug/treatment/device being shown to work and not need further testing

• Decisions made by local regulatory/health authorities.

**15 What happens when the research project ends?**

At the conclusion of this research project FMT may not continue to be available for participants with Crohn’s disease. If this is the case, participants can discuss with study doctors about the other treatment options currently available including steroid based therapy, immunomodulators, biological drugs or surgery. Participants may also be suitable for consideration for other clinical trials of new drug therapies.

We will keep in touch with you yearly by phone for up to 5 years after your treatment to collect long term data on your health for a long-term database FMT recipient outcomes.

Participants will be kept informed via email of study results and publications. Participants will have the contact details for our study coordinators who they can contact at any time for an update about the success of the project.

**Part 2 How is the research project being conducted?**

**16 What will happen to information about me?**

By signing the consent form, you consent to the study doctor and relevant research staff collecting and using personal information about you for the research project. Any information obtained in connection with this research project that can identify you will remain confidential. Data collected will be re-identifiable (coded). Only study investigators will be able to identify participants based on the data collected. Details about this coding will be strictly confidential and not disclosed to anyone outside of the study team, including our scientific collaborators. Only clinical study investigators will be able to identify participants based on the data collected. Details about this coding will be strictly confidential and not disclosed to anyone outside of the St Vincent’s Hospital study team.

Clinical information, which forms care for the management of a patient’s Crohn’s disease will be placed on the scanned medical records at St Vincent’s Hospital. Electronic and hard copies will be kept within the Department of Gastroenterology and with medical records. Study related data will be coded, and kept in a locked filing cabinet. The locked filing cabinet will be kept within a room with a locked door within the Department of Gastroenterology.

Information collected via your smart phone using the MIRO app will not be stored or linked with any data that may identify you, until the data has been transferred to the REDCap database.

All electronic data will be kept on password protected folders within the St Vincent’s Hospital IT system. The database containing the information will be REDCap (a data collection tool) which is kept on a secure server within the University of Melbourne.

Only investigators will have access to the electronic and hard copies. All copies will be kept for a minimum of 15 years.

Your information will only be used for the purpose of this research project and it will only be disclosed with your permission, except as required by law.

Information about you may be obtained from your health records held at this and other health services for the purpose of this research. By signing the consent form, you agree to the study team accessing health records if they are relevant to your participation in this research project.

It is anticipated that the results of this research project will be published and/or presented in a variety of forums. In any publication and/or presentation, information will be provided in such a way that you cannot be identified.

Information about your participation in this research project may be recorded in your health records.

In accordance with relevant Australian and Victorian privacy and other relevant laws, you have the right to request access to your information collected and stored by the research team. You also have the right to request that any information with which you disagree be corrected. Please contact the study team member named at the end of this document if you would like to access your information.

Any information obtained for the purpose of this research project *and for the future research described in Section 16* that can identify you will be treated as confidential and securely stored. It will be disclosed only with your permission, or as required by law.

**17 Complaints and compensation**

If you suffer any injuries or complications as a result of this research project, you should contact the study team as soon as possible and you will be assisted with arranging appropriate medical treatment. If you are eligible for Medicare, you can receive any medical treatment required to treat the injury or complication, free of charge, as a public patient in any Australian public hospital.

If you have any complains about your treatment as part of this clinical trial then please contact the study team as soon as possible. All complains will be handled seriously by the senior members of the study team. When necessary mediation will be provided. If this is not satisfactory then complains should be directed to the Office of the Australian Information Commissioner.

**18 Who is organising and funding the research?**

This research project is being conducted by a research team from St Vincent’s Hospital Melbourne who receive salaries from St Vincent’s Hospital Melbourne, The National Health and Medical Research Council of Australia and The University of Melbourne. Investigators from the Queen Elizabeth Hospital (Dr’s Sam Costello and Robert Bryant) and Dr Sam Forster are shareholders in BiomeBank. However, beyond provision of stool for FMT to meet study needs, their involvement in the study is academic.

St Vincent’s Hospital Melbourne is purchasing FMT from BiomeBank for this study. BiomeBank may benefit financially from this research project if, for example, the project assists BiomeBank to obtain approval for a new drug.

By taking part in this research project you agree that samples of your blood and faeces (or data generated from analysis of these materials) will be provided to St Vincent’s Hospital Melbourne, University of Queensland, BiomeBank or the Hudson Institute of Medical Research who may directly or indirectly benefit financially from your samples or from knowledge acquired through analysis of your samples.

You will not benefit financially from your involvement in this research project even if, for example, your samples (or knowledge acquired from analysis of your samples) prove to be of commercial value to St Vincent’s Hospital Melbourne, University of Queensland, BiomeBank or the Hudson Institute of Medical Research.

In addition, if knowledge acquired through this research leads to discoveries that are of commercial value to St Vincent’s Hospital Melbourne, University of Queensland, BiomeBank or the Hudson Institute of Medical Research, the study doctors or their institutions, there will be no financial benefit to you or your family from these discoveries.

No member of the research team at St Vincent’s Hospital Melbourne or at the Queen Elizabeth Hospital, Adelaide will receive a personal financial benefit from your involvement in this research project (other than their ordinary wages).

**19 Who has reviewed the research project?**

All research in Australia involving humans is reviewed by an independent group of people called a Human Research Ethics Committee (HREC). The ethical aspects of this research project have been approved by the HREC of St Vincent’s Hospital Melbourne.

This project will be carried out according to the *National Statement on Ethical Conduct in Human Research (2007)*. This statement has been developed to protect the interests of people who agree to participate in human research studies.

St Vincent’s Hospital Melbourne has given approval for this research to be carried out at this institution and will be responsible for supervising the standard of care provided as part of this project.

**20 Further information and who to contact**

The person you may need to contact will depend on the nature of your query.

If you want any further information concerning this project or if you have any medical problems which may be related to your involvement in the project (for example, any side effects), you can contact the principal study doctors or clinical contact person at your site.

For matters relating to research at the site at which you are participating, the details of the local site complaints person are:

**Complaints contact person**

If you have any complaints about any aspect of the study or the way in which it is being conducted you may contact the Patient Liaison Officer at your study site.

Patient Liaison Officer contact: You will need to tell the Patient Liaison Officer the name of the person who is noted above as principal investigator.

If you have any complaints about any aspect of the project, the way it is being conducted or any questions about being a research participant in general, then you may contact:

| Position |  |
| --- | --- |
| Telephone |  |
| Email |  |

**Research Participant Rights:**

If you have any questions about your rights as a research participant, then you may contact

the Executive Officer at your study site.

| Reviewing HREC Name | St. Vincent’s Hospital Melbourne HREC |
| --- | --- |
| Position | HREC Executive Officer |
| Telephone | (03) 9231 **6970** |
| Email | [Research.ethics@svhm.org.au](about:blank) |

**Consent Form -** *Adult providing own consent*

| **Title** | Faecal Microbiota Transplantation (FMT) In Crohn’s Disease: The MIRO II (Microbial Restoration) Study |
| --- | --- |
| **Short Title** | The MIRO II Study |
| **Project Sponsor** | St Vincent’s Public Hospital, Melbourne |
| **Coordinating Principal Investigator/ Principal Investigator** | Prof. Michael Kamm |
| **Location** |  |

**Declaration by Participant**

I have read the Participant Information Sheet or someone has read it to me in a language that I understand.

I understand the purposes, procedures and risks of the research described in the project.

I give permission for my doctors, other health professionals, hospitals or laboratories outside this hospital to release information to St Vincent’s Public Hospital, Melbourne concerning my disease and treatment for the purposes of this project. I understand that such information will remain confidential.

I consent to the storage and use of blood, stool and tissue samples taken from me for use, as described in the relevant section of the Participant Information Sheet, for:

• This specific research project

• Other research that is closely related to this research project

By signing this consent section, I agree to the use of tissue samples obtained previously from my colonoscopy for the purposes of additional histologic assessment.

By signing this consent section, I agree to the use of my blood/tissue samples for genetic testing, as outlined in the relevant Section of the Participant Information Sheet.

I understand that, if I decide to discontinue the study treatment, I may be asked to attend follow-up visits to allow collection of information regarding my health status. Alternatively, a member of the research team may request my permission to obtain access to my medical records for collection of follow-up information for the purposes of research and analysis.

I have had an opportunity to ask questions and I am satisfied with the answers I have received.

I freely agree to participate in this research project as described and understand that I am free to withdraw at any time during the study without affecting my future health care.

I understand that I will be given a signed copy of this document to keep.

|  | | | | | | |
| --- | --- | --- | --- | --- | --- | --- |
|  | Name of Participant (please print) | |  |  |  |  |
|  | | | | | | |
|  | Signature |  | | Date |  |  |
|  | | | | | | |

|  | | | | | | |
| --- | --- | --- | --- | --- | --- | --- |
|  | Name of Witness* to Participant’s Signature (please print) | |  | | |  |
|  | | | | | | |
|  | Signature |  | | Date |  |  |
|  | | | | | | |

* Witness is not to be the investigator, a member of the study team or their delegate. In the event that an interpreter is used, the interpreter may not act as a witness to the consent process. Witness must be 18 years or older.

**Declaration by Study Doctor/Senior Researcher^†^**

I have given a verbal explanation of the research project, its procedures and risks and I believe that the participant has understood that explanation.

|  | | | | | | |
| --- | --- | --- | --- | --- | --- | --- |
|  | Name of Study Doctor/  Senior Researcher^†^ (please print) | |  | | |  |
|  | | | | | |  |
|  | Signature |  | | Date |  |  |
|  | | | | | | |

^†^ A senior member of the research team must provide the explanation of, and information concerning, the research project.

Note: All parties signing the consent section must date their own signature

**Form for Withdrawal of Participation -** *Adult providing own consent*

| **Title** | Faecal Microbiota Transplantation (FMT) In Crohn’s Disease: The MIRO II (Microbial Restoration) Study |
| --- | --- |
| **Short Title** | The MIRO II Study |
| **Project Sponsor** | St Vincent’s Public Hospital, Melbourne |
| **Coordinating Principal Investigator/ Principal Investigator** | Professor Michael Kamm |
| **Location** |  |

**Declaration by Participant**

I wish to withdraw from participation in the above research project and understand that such withdrawal will not affect my routine treatment, my relationship with those treating me or my relationship with St Vincent’s Public Hospital, Melbourne

|  | | | | | | |
| --- | --- | --- | --- | --- | --- | --- |
|  | Name of Participant (please print) | |  |  |  |  |
|  | | | | | | |
|  | Signature |  | | Date |  |  |
|  | | | | | | |

*In the event that the participant’s decision to withdraw is communicated verbally, the Study Doctor/Senior Researcher will need to provide a description of the circumstances below.*

|  |
| --- |

**Declaration by Study Doctor/Senior Researcher^†^**

I have given a verbal explanation of the implications of withdrawal from the research project and I believe that the participant has understood that explanation.

|  | | | | | | |
| --- | --- | --- | --- | --- | --- | --- |
|  | Name of Study Doctor/  Senior Researcher^†^ (please print) | |  | | |  |
|  | | | | | |  |
|  | Signature |  | | Date |  |  |
|  | | | | | | |

^†^ A senior member of the research team must provide the explanation of and information concerning withdrawal from the research project.

Note: All parties signing the consent section must date their own signature.
