# Supplementary material for: Faecal microbiota transplantation in Crohn’s disease: an Australian randomised placebo-controlled trial protocol
Source: BMJ Open. 2025 Apr 19;15(4):e094714. doi: 10.1136/bmjopen-2024-094714 (PMC12010309; doi:10.1136/bmjopen-2024-094714)
Supplement: Supplementary file 3 [file bmjopen-2024-094714.reviewer_comments.pdf]

PEER REVIEW HISTORY

BMJ Open publishes all reviews undertaken for accepted manuscripts. Reviewers are asked to complete a checklist review form and are provided with free text boxes to elaborate on their assessment. These free text comments are reproduced below.

ARTICLE DETAILS

Title (Provisional)

Faecal Microbiota Transplantation in Crohn’s Disease: An Australian Randomised Placebo-Controlled Trial Protocol

Authors

Fehily, Sasha R; Wright, Emily K.; Basnayake, Chamara; Wilson-O'Brien, Amy; Stanley, Anna; Marks, Elise; Russel, Erin; Hamilton, Amy; Bryant, Robert V; Costello, Sam; Kamm, Michael A

VERSION 1 - REVIEW

**Reviewer**	**1**
**Name**	**Qiao, Yuqi**
**Affiliation**	**Shanghai Jiao Tong University School of Medicine Affiliated Renji Hospital**
**Date**	**15-Dec-2024**
**COI**	**None**

This study design is a randomized, double-blind, placebo-controlled clinical trial aimed at evaluating the efficacy and safety of fecal microbiota transplantation (FMT) combined with existing treatments in inducing and maintaining remission in patients with active Crohn's disease. Below are some discussions and recommendations.

1. Sample size calculation: The manuscript mentions that the sample size is estimated based on limited data and experience. Ensure that the sample size calculation considers the expected effect size, statistical power, significance level, and anticipated dropout rate.

2. Use of antibiotics: Administering antibiotics prior to FMT may enhance the engraftment of FMT, but this is a controversial approach. It is important to monitor whether this affects the long-term balance of the microbiota. There are questions about whether antibiotics themselves influence the treatment of Crohn's disease, and potential risks associated with antibiotic use need to be addressed. Informed consent should clearly explain the rationale and risks associated with the use of antibiotics.

3. Dietary optimization phase: Adherence to the dietary regimen might be difficult to accurately assess, and patients may struggle to adhere long-term. The manuscript does not detail how adherence will be ensured.

4. Patient diversity: The manuscript mentions that patients will be recruited from two specialized IBD centers, but it does not specify how diversity in the patient population will be ensured, including factors such as race, age, and disease subtypes.

5. Long-term follow-up: Crohn's disease requires long-term maintenance therapy. The manuscript does not clearly outline plans for long-term follow-up, only mentioning a 44-week treatment maintenance phase.

VERSION 1 - AUTHOR RESPONSE

Reviewer: 1

1. Sample size calculation: The manuscript mentions that the sample size is estimated based on limited data and experience. Ensure that the sample size calculation considers the expected effect size, statistical power, significance level, and anticipated dropout rate.

> **RESPONSE:** The sample size calculation considers the expected effect size, statistical power, significance level based on data from the few controlled trials evaluating FMT in Crohn's disease and the pharmaceutical registration trials in Crohn's disease. Anticipated dropout rate was also incorporated into the sample size calculation.
>
> **Page 6:** "The study aims to enrol 120 patients with a goal of 96 patients completing the induction phase. This based on registration studies evaluating induction of remission with anti-TNF therapy in Crohn's disease (cA2 study(30) and CLASSIC I study(31)). Differences ranging from 24% to 29% were achieved (remission rates in intervention group of 33% - 36%; remission rates in placebo group of 4% - 12%). In additiontwo small controlled trials evaluating FMT in Crohn's disease reported remission rates in the intervention group of 36% and 44% weeks 8 and 10, respectively (32, 33).
>
> The predicted proportion of patients achieving response is 60% in the FMT group and 30% in the control group (estimated delta of 30%), based on a double sided alpha value of 0.05. This also takes into account 20% attrition in each group, to achieve a non-drop out number of 96 patients completing the induction phase of the study."

2. Use of antibiotics: Administering antibiotics prior to FMT may enhance the engraftment of FMT, but this is a controversial approach. It is important to monitor whether this affects the long-term balance of the microbiota. There are questions about whether antibiotics themselves influence the treatment of Crohn's disease, and potential risks associated with antibiotic use need to be addressed. Informed

consent should clearly explain the rationale and risks associated with the use of antibiotics.

RESPONSE: The literature supports pre-FMT antibiotics to improve engraftment as well as the therapeutic response from FMT. In addition, antibiotics will be given to both placebo control and active treatment groups, leaving FMT as the only variable.

Page 13: The following reference has been added: *Podlesny D, Durdevic M, Paramsothy S, Kaakoush NO, Högenauer C, Gorkiewicz G, Walter J, Fricke WF. Identification of clinical and ecological determinants of strain engraftment after fecal microbiota transplantation using metagenomics. Cell Reports Medicine. 2022 Aug 16;3(8).*

"Animal and human studies have demonstrated improved donor engraftment when using combination broad spectrum antibiotic therapy."

We have not included risks from antibiotics in the consent form, as they were not regarded as substantial. Further, antibiotics are often used in the treatment of Crohn's disease and are often a routine part of management

3. Dietary optimization phase: Adherence to the dietary regimen might be difficult to accurately assess, and patients may struggle to adhere long-term. The manuscript does not detail how adherence will be ensured.

RESPONSE: We agree with this comment, which is why the dietary modification is recommended to all patients in the study, rather than a prescribed regimen. The dietary advice serves to support the preservation of the improved microbiota and enhance the overall effectiveness of the intervention. We have, however, included compliance assessment at the end of the induction and maintenance phases. This will allow us to assess the impact of dietary intervention adherence on outcomes.

Page 13: "We included compliance assessment at the end of the induction and maintenance phases. This will allow us to assess the impact of dietary intervention adherence on outcomes."

4. Patient diversity: The manuscript mentions that patients will be recruited from two specialized IBD centres, but it does not specify how diversity in the patient population will be ensured, including factors such as race, age, and disease subtypes.

RESPONSE: Patient diversity will be ensured by the recruitment process. The two specialized IBD centres serve large and diverse catchment areas, encompassing individuals with varying racial, ethnic, and socioeconomic backgrounds. Recruitment will include referrals from gastroenterologists and general practitioners, covering both private practices and public clinics across

two Australian states. Additionally, we will include referrals from rural centres to further ensure diversity in the patient population.

Page 4: "Patient diversity will be ensured by the recruitment process. The study will be conducted at two Australian secondary and tertiary specialist IBD centres that serve large and diverse catchment areas. Patients will be referred by gastroenterologists from within, and outside, these two specialist centres, including from both private practices and public clinics across two Australian states. Study commencement was May 2022 with intended conclusion in April 2026."

5. Long-term follow-up: Crohn's disease requires long-term maintenance therapy. The manuscript does not clearly outline plans for long-term follow-up, only mentioning a 44-week treatment maintenance phase.

RESPONSE: Clinical data will be collected out to 5 years as outlined in the patient informed consent form. The details have not been protocolised as they are yet to be determined.

Page 7: "Clinical data will be collected out to 5 years."

6. Additional amendments

Endoscopic assessment

Page 9: Endoscopic images will not be assessed by a blinded central reader.

Emergency requirement for unblinding

Page 9: "In the event that a patient needs to be unblinded for medical care, a 24 hour contact number is available for immediate unblinding."

Early Discontinuation/Withdrawal/Treatment Failure

Page 10: "Patients may be withdrawn from the study if any of the following occurs:

- The patient requests withdrawal from the study.
- Fails to attend one of the major study visits.
- Treatment Failure, defined as:
- Clinical recurrence: symptoms typical of Crohn's disease, necessitating unscheduled endoscopic or surgical intervention or addition of new drug therapy), associated with confirmed active disease.
- Failed corticosteroid withdrawal.
- Serious adverse events or complications necessitating cessation of therapy.
- Pregnancy.

The investigator can also decide to withdraw a patient from the study if he/she considers it as necessary (e.g. non-respect of at least one of the selection criteria that becomes known after inclusion).

At the time of withdrawal/discontinuation, patients will undergo a disease activity assessment including CRP, calprotectin, sonographic and endoscopic assessment if possible."

Data monitoring

Page 11: "The *audit* plan will involve 100% manual verification checks on a *10*% random sample of participant files."

Subgroup analyses

Page 12: "Subgroup analyses will include outcomes in the upper and lower gut delivery cohorts, and many of the patient demographic variables."

Data sharing

Page 12: "Data can be accessed by request to the principal investigator."

Biospecimen collection

Page 8: "Biological specimens will be collected in line with the study protocol and standard study procedures."

Funding statement

Page 15: "No funder had any role in study or protocol design, or data collection, management or analysis."
